# Analysis of the Clinical Value of Delphian Lymph Node Metastasis in Papillary Thyroid Carcinoma

**DOI:** 10.1155/2022/8108256

**Published:** 2022-06-10

**Authors:** Qiumei Zuo, Xin Chen, Jingyu Yang, Shuang Qiu, Yuan Zhao, Jianwei Sun

**Affiliations:** ^1^Department of Thyroid and Breast Surgery, The First People's Hospital of Yunnan Province, Kunming, China; ^2^The Affiliated Hospital of Kunming University of Science and Technolgy, Kunming, Yunnan 650500, China; ^3^Department of Oncology, Puer People's Hospital, Puer, Yunnan 665000, China

## Abstract

**Purpose:**

Delphian lymph node (DLN) is often involved in metastasis of malignant head and neck tumors. This study evaluates the predictive utility of the DLN and the clinicopathological factors related to DLN metastasis in individuals suffering from papillary thyroid carcinoma (PTC). *Patients and Methods*. A retrospective analysis was made on 969 PTC patients enrolled from 2017 to 2021. Among these patients, 522 PTC patients are DLN positive and 447 are negative. Comparisons of clinicopathological characteristics between the DLN-positive and DLN-negative patients were made.

**Results:**

The DLN was detected in 53.9% (522/969) cases, and DLN metastasis occurred in 20.3% (106/522) cases. The independent predictors of DLN metastasis (DLNM) include tumor size >1 cm, tumor located in the upper third thyroid or isthmus, central lymph node metastasis (CLNM), and lateral lymph node metastasis (LLNM). DLN-positive individuals exhibited a higher incidence and the number of CLNM, contralateral CLNM (CCLNM), and LLNM as compared to DLN-negative patients. Whether it is cN0 or cN+, the CLNM incidence was increased among DLN-positive patients as compared to that of DLN-negative patients.

**Conclusions:**

Positive DLN indicated an increased rate and number of metastases in the cervical lymph nodes. Intraoperative rapid freezing is recommended to assess the status of the DLN, and careful assessment of cervical lymph nodes is warranted when the DLN is involved to implement an appropriate surgical approach.

## 1. Introduction

With the increasing popularization of head and neck ultrasonography (US) in thyroid cancer screening, the number of thyroid cancer patients has increased [1]. The most prevalent thyroid cancer type (>90%) is papillary thyroid carcinoma (PTC) [2, 3]. PTC is a kind of indolent cancer. Its 5-year overall survival (OS) rate can be up to 97%, but 20–80% of early-stage cancer patients exhibit lymph node metastasis, which augments the risk of regional recurrence and affects the overall quality of life [4–7]. The most important method for preoperative assessment of cervical lymph nodes is US, but the sensitivity of preoperative US to detect lateral lymph nodes is only about 30% [8]. Therefore, finding a reliable index to accurately assess the lymph nodes is very important for guiding the scope of thyroid surgery. The Delphian lymph node (DLN), named in honor of the Delphian oracle of ancient Greece [9], is also referred to as the prelaryngeal lymph node and is a central cervical lymph node (CLN), as are the paratracheal and pretracheal nodes. The DLN was first realized in throat and hypopharyngeal cancers, which predicts elevated mortality and recurrence rates. It has received more and more attention because of its unique anatomical position [10, 11]. Recently, some researchers have explored the clinical relevance of the DLN in PTC, demonstrating that DLN metastasis (DLNM) is of a prognostic value in cervical and lateral lymph nodes [12–14]. However, these studies had small sample sizes or reported different clinicopathological factors associated with DLNM. In the context of limited studies, the clinical significance of DLN remains controversial.

Here, we explore risk factors that affect DLN metastasis using the large sample size and clarify the correlation between the DLN and cervical lymph nodes to provide a basis for choosing an appropriate scope of surgery for PTC patients.

## 2. Materials and Methods

### 2.1. Patients

Clinical and pathological data from 969 PTC patients who underwent the first operation in the Sun Jianwei Medical Group of the Thyroid and Breast Surgery Department of the First People's Hospital of Yunnan Province from January 2017 to May 2021 were reviewed. The Ethics Committee of Yunnan First People's Hospital approved this analysis, and all patients signed informed consents. Of the 969 included patients, 522 had DLNs, and the remaining 447 had adipose tissue in the prelaryngeal area. The information collected was as follows: sex, age, tumor size, bilaterality, multifocality, the number of tumors, extrathyroidal extension (ETE), central and lateral lymph nodes of the neck, pathologic types, Hashimoto's thyroiditis, and cancerous nodules. In the case of multifocality, the largest lesion was measured when judging the tumor size and location.

### 2.2. Surgery

**Figure 1:**
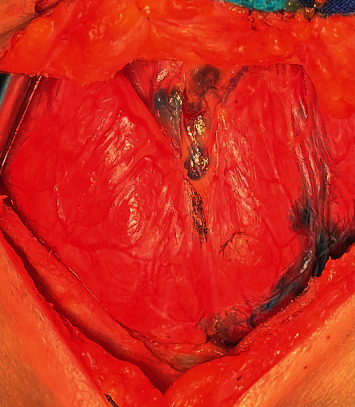
Intraoperative lymph node tracing following the injection of carbon nanoparticles.

Bilateral PTC undergoes total thyroidectomy, and patients with unilateral PTC undergo total/near-total thyroidectomy or lobectomy and isthmus resection. Total thyroidectomy may be considered in cases in which unilateral PTC has more than one of the following: tumor size >4 cm, one lobe with multifocality, ETE, or distant metastasis (as per the Chinese Thyroid Association guidelines). Central lymph node dissection (CLND) was routinely conducted in all patients on the affected side, while contralateral CLND was performed when the central lymph node was suspected by preoperative imaging or intraoperative examination. Ipsilateral therapeutic lateral lymph node dissection (LLND) was conducted when suspected cervical lymph nodes were detected by preoperative physical examination, imaging (ultrasound, CT, MRI, and/or PET-CT), fine-needle aspiration cytology (FNAC), and/or intraoperative visual examination. LLND resection included lymph nodes at levels II–V. During this operation, the bilateral thyroid gland lobes were injected using carbon nanoparticles (Chongqing Levin U.S. Pharmaceutical, China, approval number: Zhunzi H20073246), which were lymphatic tracers that can assist in tracing the DLN (Figure 1). Central lymph nodes were routinely removed. Soft fascial tissue above the thyroid isthmus in front of the thyroid and cricoid cartilage was labeled DLN, with remaining central lymph nodes (paratracheal and pretracheal) being labeled CLNs.

### 2.3. Statistical Analysis

SPSS 26.0 (IBM Analytics, USA) was used to analyze data. Qualitative data were compared via the Pearson *χ*2 test. Fisher's exact test was used for sample sizes less than 5. For continuous variables, an independent sample *t*-test is used. Multivariate logistic regression was conducted to estimate DLNM risk factors for all variables significant in univariate analysis. *P* < 0.05 was the significance threshold.

## 3. Results

### 3.1. General Characteristics of DLN

A total of 969 patients were included in this analysis, 522 (53.9%) had the DLN detected. The average number of DLNM was 2, of which 106 (20.3%) had DLNM (Table 1). It is worth noting that 17 (1.7%) patients exhibited only DLNM without central lymph node metastasis. Four cases (10%) showed jump metastasis (LLN positive and CLN negative). Among the 522 patients with PTC, 408 underwent total thyroidectomy and 114 underwent lobectomy plus isthmus resection. According to the above standards, all patients received CLND and 46 patients received LLND (Table 2).

### 3.2. Clinical and Pathological Features of DLNM

The clinicopathological characteristics of DLN-positive and DLN-negative groups were initially compared via univariate analysis (Table 3). Males were more common among DLN-positive patients in comparison to DLN-negative patients (27.4% vs. 17.1%, *P*=0.016). The proportion of tumor size >1 cm (67.0% vs. 22.6%, *P* < 0.001) and the tumor location in the upper 1/3 and isthmus (41.8% vs. 27.0%, *P*=0.003) was more common among those exhibiting DLN positivity. There was a significant correlation between DLNM and ETE (21.7% vs. 7.5%, *P* < 0.001), close to the envelope (5.7% vs. 1.4%, *P*=0.026), CLNM (84.0% vs. 29.8%, *P* < 0.001), and LLNM (30.2% vs. 2.2%, *P* < 0.001). The average maximum size of metastatic lymph nodes was increased among DLN-positive patients relative to those that were DLN-negative (2.71 ± 1.07 vs. 2.12 ± 0.75, *P* < 0.001). After 0.5 to 5 years of follow-up, four patients (0.77%) had a locoregional recurrence. Locoregional recurrence was more likely to occur with the DLN than without the DLN (2.8% vs. 0.2%, *P*=0.028). There was no statistically significant difference between the two groups in the following variables: age (<55 or ≥ 55 years), multifocality, bilaterality, Hashimoto's thyroiditis, and cancerous nodule.

Through univariate analysis, variables associated with DLN-positive risk factors were screened out for multivariate analysis. Tumor size >1 cm (OR = 3.051, *P* < 0.001), the tumor located in the upper 1/3 or isthmus (OR = 0.402, *P*=0.002), CLNM (OR = 0.121, *P* < 0.001), and LLNM (OR = 0.151, *P* < 0.001) are independent risk factors associated with DLNM (Table 4).

### 3.3. Correlations between DLNM and CLN

In this study, 372 patients underwent unilateral CLND and 150 patients underwent bilateral CLND. In patients with unilateral CLND, the proportion and the number of CLNM were higher among DLN-positive individuals relative to those that were DLN-negative (79.0% vs. 27.1%, *P* < 0.001; 3.69 ± 2.38 vs. 2.06 ± 1.64, *P* < 0.001). The same situation also appeared in bilateral CLND (93.0% vs. 37.4%, *P* < 0.001; 5.90 ± 4.62 vs. 2.95 ± 3.35, *P*=0.002). Further analysis revealed that compared with the DLN-negative group, the incidence of CLNM was greater than 5 in the DLN-positive group.

The DLN-positive group was increased relative to DLN-negative patients (unilateral: 28.6% vs. 7.1%, *P*=0.01; bilateral: 47.5% vs. 10.0%, *P* < 0.001) (Table 5).

It is interesting to note that there were 68 cases of total thyroidectomy plus bilateral CLND and postoperative pathological confirmation of unilateral PTC, including 20 cases in the DLN-positive group and 48 cases in the DLN-negative group. The incidence of contralateral CLNM (CCLNM) in the DLN-positive group was higher than that in the DLN-negative group (50.0% vs. 6.3%, *P* < 0.001). Similarly, for the ipsilateral CLN metastasis, the DLN-positive group was also higher than the DLN-negative group (95.0% vs. 20.8%, *P* < 0.001) (Table 6).

### 3.4. Correlation between DLNM and LLN

Forty-six patients underwent LLND, including 42 cases of unilateral LLND with 36 cases of LLNM and 4 cases of bilateral LLND with 4 cases of LLNM. Among DLN-positive patients, the incidence of LLNM was higher as compared to DLN-negative patients (93.9% vs. 69.2%, *P*=0.045). However, there were no differences in LLNM numbers between the DLN-positive and DLN-negative groups (6.23 ± 4.50 vs. 3.89 ± 2.09, *P*=0.142) (Table 7).

### 3.5. Correlation between DLNM and Clinical N Stage

Table 8 shows the CLNM incidence in the clinical-stage N. cN0: the incidence of CLNM was 32.1%. The incidence of CLNM was significantly elevated among DLN-positive patients relative to those that were DLN-negative (73.5% vs. 26.6%, *P* < 0.001; 18.4% vs. 6.8%, *P*=0.011) in the patients at cN0 or T3/T4 tumors. In cN + patients, the incidence of CLNM was 93.0% in the DLN metastasis group and 55.3% in those without the DLN metastasis group (*P* < 0.001).

## 4. Discussion

This analysis retrospectively examined the real-world data of 522 PTC patients. The clinicopathological characteristics pertaining to lymph node metastasis were systematically and comprehensively evaluated. DLN metastases in PTC patients were related to tumor aggressiveness and indicated CLN and LLN metastasis.

DLNM is widely regarded as an indicator of a poor prognosis of head and neck malignancies, particularly PTC. Isaacs et al. found that the DLN was more accurate in predicting cervical lymph node metastasis than paratracheal and pretracheal lymph nodes [15]. In previous studies, the rate of DLN metastasis in PTC patients was .9%–25.4% [16, 17]. In our study, the rate of DLNM was 20.3%, which aligns with other studies. Cervical lymph node metastasis can predict recurrence. Those individuals with more or larger lymph node metastasis have a significantly higher risk of recurrence [18–20]. In our study, the numbers and rates of CLNMs and LLNMs were elevated in DLNM patients than those without such metastases and the proportion of metastatic CLNs greater than 5 was higher. Moreover, metastatic LNs were larger on average among DLN-positive patients in comparison to those that were DLN-negative. Sugitani et al. also found lymph node metastases larger than 3 cm to be related to disease-specific survival among individuals with PTC [21]. These studies suggest that if DLN metastasis is suspected during surgery, surgeons must fully assess the CLN and LLN status before deciding on the scope of surgery.

The previous study has indicated that DLNM is closely related to tumor size, location, ETE, multifocality, bilaterality, central and lateral cervical lymph node metastasis, and more [22–24]. The clinicopathological factors related to DLN metastasis were inconsistent in previous reports. Cancer nodules present in lymph nodes of PTC patients are associated with distant metastasis and worse survival outcomes [25]. Here, the appearance of cancer nodules did not differ as a function of the DLN status. Males harboring larger tumors are at a higher risk of DLNM development, and DLNM suggests a higher risk of ETE and close to the capsule and the high incidence of CLNM and LLNM. In addition, tumor size >1 cm, located in the upper 1/3 and isthmus, CLNM, and LLNM were independently associated with DLNM risk. Chai et al. reported that the location of tumors in DLN-positive patients is more likely to be in the isthmus or upper 1/3 of the tumor [26]. Our research results are consistent with their finding. This finding indicates that the DLN mainly accepts lymphatic reflux from the upper-middle thyroid and isthmus. Tumors in the superior lobe of the thyroid are more prone to metastasize to the lateral lymph nodes [27, 28]. Skip metastasis rates range from 6.8 to 27.8% in PTC patients exhibiting LLNM [29, 30]. Here, the incidence of skip metastasis was 10%. These results suggest that skip metastases should be noted when tumors are located in the superior pole of the thyroid lobe, though the overall rate is low.

There is still controversy about prophylactic CLND for individuals with clinical lymph node-negative (cN0) disease. CLND should be performed for T3 or T4 tumors in individuals at high risk but should be avoided for low-risk patients who harbor T1 or T2 tumors. Fraser et al. found that only 14.7% of the cases were cN1, but 56.8% of the patients had CLNM [27]. Xue et al. also reported that the incidence of CLNM was 45.85% (713/1555), and males, age ≤45 years, ETE, and tumor size >1 cm were independently associated with the risk of CLNM among cN0 patients [31]. In our study, whether it was cN0 or cN + patients, CLNM rates in the DLN-positive patients were greatly increased relative to those in DLN-negative individuals (73.5% vs. 26.6%, *P* < 0.001; 93.0% vs. 55.3%, *P* < 0.001). In addition, Chen et al. determined that DLNM was independently predictive of CCLNM in unilateral PTC by multivariate analysis [32]. In this study, 68 unilateral PTC patients underwent total thyroidectomy and bilateral CLND. Rates of CCLNM were significantly elevated among DLN-positive individuals in comparison to those that were DLN-negative. During thyroidectomy, the DLN is the first node to be encountered and is easy to remove. Based on these findings, performing a cytopathologic examination of DLN intraoperatively is highly recommended, and unilateral or bilateral CLND is required when DLN metastasis is detected. Furthermore, cervical lymph node metastasis was not found in preoperative imaging examination and intraoperative exploration in 28%–33% of patients who were diagnosed with PTC [33], but lymph node metastasis was confirmed pathologically after preventive lymph node dissection, which changes the TNM stage and postoperative treatment plan of PTC. Prophylactic central neck dissection in patients with low-risk tumors can reduce the recurrence rate. However, some scholars do not support routinely preventive central neck dissection because it increases the risk of patients with parathyroidism, transient or permanent recurrent laryngeal nerve injury, and chylous leakage.

Many studies have shown that the DLN can also predict LLNM in PTC patients [9, 28, 34, 35]. Here, DLNM was found to be closely associated with LLNM, with DLNM suggesting higher LLNM incidence rates and numbers. In addition, DLNM is more common when LLNM occurs, particularly in bilateral LLNM patients. As N1b corresponds to a high risk of disease recurrence [36], when there is clinical N1b disease, intraoperative assessment of DLN should be performed with caution. If necessary, DLN-positive patients should have all possible LLNs fully resected, including lymph nodes at levels II-V. Patients who have not undergone LLND should be carefully monitored after surgery.

There are some limitations to this analysis. First, this is a retrospective study, which may have potential selection bias. Second, the small number of LLND cases may lead to bias in our results. Third, the association between DLN and lymphatic vascular infiltration was not examined as the pathology department did not report lymphatic vascular infiltration. PTC is an indolent cancer, and the clinical significance of DLN warrants further verification by longer follow-up.

## 5. Conclusions

DLN metastasis in PTC patients is an indicator of a poor prognosis. Intraoperative rapid freezing for the DLN is highly recommended during thyroidectomy. CLND or even CCLND should be performed when DLNM is confirmed. Patients who have not undergone lymph node dissection should be followed up closely after surgery.

## Figures and Tables

**Table 1:** Delphian lymph node detection and rate of metastasis.

Characteristics	Value
DLN detection	53.9% (522/969)
DLN metastasis	20.3% (106/522)
Mean DLN detected (range)	2.39 ± 1.50 (1–9)
Mean DLN metastases (range)	1.65 ± 1.02 (1–6)
Skip metastasis	10% (4/40)

DLN, Delphian lymph node; skip metastasis, LLN positive, and CLN negative.

**Table 2:** The extent of surgery in DLN-positive and DLN-negative groups.

Extent of surgeries	DLN-positive (*n* = 106)	DLN-negative (*n* = 416)	Total
Lobectomy plus isthmectomy			114
Ipsilateral CLND	15	99	
Total thyroidectomy			408
Unilateral CLND	34	202	
Unilateral CLNDand LLND	13	8	
Bilateral CLND	24	102	
Bilateral CLND and unilateral LLND	17	4	
Bilateral CLND and LLND	3	1	

DLN, Delphian lymph node; LLND, lateral lymph node dissection; CLND, central lymph node dissection.

**Table 3:** DLN clinical and pathological features in univariate analysis.

Characteristics	DLN-positive *n* (%)	DLN-negative *n* (%)	*P* value
Sex			0.016
Male	29 (27.4)	71 (17.1)	
Female	77 (72.6)	345 (82.9)	
Age			0.053
≥55 years	97 (91.5)	350 (84.1)	
<55 years	9 (8.5)	66 (15.9)	
Tumor size			<0.001
≤1 cm	35 (33.0)	322 (77.4)	
>1 cm	71 (67.0)	94 (22.6)	
Multifocality			0.363
Single	85 (80.2)	349 (83.9)	
Multiple	21 (19.8)	67 (16.1)	
Bilaterality			0.129
Bilateral	74 (69.8)	320 (76.9)	
Unilateral	32 (30.2)	96 (23.1)	
Tumor location			0.003
Upper	35 (33.3)	98 (23.6)	
Middle	41 (38.7)	162 (38.9)	
Low	21 (19.8)	142 (34.1)	
Isthmus	9 (8.5)	14 (3.4)	
ETE			<0.001
Yes	23 (21.7)	31 (7.5)	
No	83 (78.30)	385 (92.5)	
Hashimoto's thyroiditis			0.060
Yes	18 (17.0)	107 (25.7)	
No	88 (83.0)	309 (74.3)	
Close to capsule			0.026
Yes	6 (5.7)	6(1.4)	
No	100 (94.3)	410 (98.6)	
CLNM			<0.001
Yes	89 (84.0)	124 (29.8)	
No	17 (16.0)	292 (70.2)	
LLNM			<0.001
Yes	32 (30.2)	9 (2.2)	
No	74 (69.8)	407 (97.8)	
Mean largest size of an LNM, cm			<0.001
Mean ± SD	2.71 ± 1.07	2.12 ± 0.75	
Cancerous nodules			1.000
Yes	1 (0.9)	4(1.0)	
No	105 (99.1)	412 (99.0)	
Locoregional recurrence			0.028
Yes	3 (2.8)	1 (0.2)	
No	103 (97.2)	415 (99.8)	

DLN, Delphian lymph node; SD, standard deviation; LLNM, lateral lymph node metastasis; CLNM, central lymph node metastasis; ETE, extrathyroidal extension.

**Table 4:** DLNM-related multivariate logistic regression analyses.

Characteristics	95% CI	OR	*P* value
Sex	0.305∼1.051	0.566	0.072
Upper or isthmus	0.224∼0.720	0.402	0.002
Close to capsule	0.123∼2.918	0.600	0.527
ETE	0.326∼1.501	0.699	0.357
Tumor size > 1 cm	1.741∼5.345	3.051	<0.001
CLNM	0.065∼0.226	0.121	<0.001
LLNM	0.063∼0.366	0.151	<0.001

DLNM, Delphian lymph node metastasis; LLNM, lateral lymph node metastasis; ETE, extrathyroidal extension; CLNM, central lymph node metastasis.

**Table 5:** Relationship between the DLN and CLNM.

	DLN-positive	DLN-negative	*P* value
*Unilateral CLND (N* *=* *372) n (%)*			
CLNM			<0.001
Yes	49 (79.0)	84 (27.1)	
No	13(21.0)	226 (72.9)	
No. of CLNM			<0.001
Mean ± SD	3.69 ± 2.38	2.06 ± 1.64	
>5	14 (28.6)	6 (7.1)	0.01
1–5	35 (71.4)	78 (92.9)	

*Bilateral CLND (N* *=* *150) n (%)*			
CLNM			
Yes	40 (93.0)	40 (37.4)	<0.001
No	3 (7.0)	67 (62.6)	
No. of CLNM			
Mean ± SD	5.90 ± 4.62	2.95 ± 3.35	0.002
>5	19 (47.5)	1 (10.0)	<0.001
1–5	21 (52.5)	36 (90.0)	

DLN, Delphian lymph node; CLND, central lymph node dissection; CLNM, central lymph node metastasis; No., number; SD, standard deviation.

**Table 6:** Ipsilateral and contralateral CLNM in 68 unilateral PTC patients with total thyroidectomy and bilateral CLND.

CLNM	DLN-positive *n* (%)	DLN-negative *n* (%)	*P* value
Ipsilateral			<0.001
Yes	19 (95.0)	10 (20.8)	
No	1 (5.0)	38 (79.2)	
Contralateral			<0.001
Yes	10 (50.0)	3 (6.3)	
No	10 (50.0)	45 (93.8)	

DLN, Delphian lymph node; CLNM, central lymph node metastasis; CLND, central lymph node dissection; PTC, papillary thyroid carcinoma.

**Table 7:** Relationship between DLN and LLNM.

	DLN-positive	DLN-negative	*P* value
LLNM			0.045
Yes	31 (93.9)	9 (69.2)	
No	2 (6.1)	4 (30.8)	
No. of LLNM			
Mean ± SD	6.23 ± 4.50	3.89 ± 2.09	0.142

DLN, Delphian lymph node; LLNM, lateral lymph node metastasis; SD, standard deviation; No., number.

**Table 8:** CLNM in cN0 or cN + PTC.

	DLN-positive	DLN-negative	Total	*P* value
*CLNM in cN0 PTC n (%)*				
CLNM				<0.001
Yes	36 (73.5)	98 (26.6)	134 (32.1)	
No	13 (26.5)	271 (73.4)	284 (67.9)	
T stage				0.011
T1/T2	40 (81.6)	344 (93.2)	384 (91.9)	
T3/T4	9 (18.4)	25 (6.8)	34 (8.1)	

*CLNM in l cN* *+* *PTC n (%)*				
CLNM				<0.001
Yes	53 (93.0)	26 (55.3)	79 (76.0)	
No	4 (7.0)	21 (44.7)	25 (24.0)	
T stage				0.900
T1/T2	48 (84.2)	40 (85.1)	88 (84.6)	
T3/T4	9 (15.8)	7 (14.9)	16 (15.4)	

DLN, Delphian lymph node; CLNM, central lymph node metastasis.

## Data Availability

The data are available from the corresponding author upon request via email (9y140257@kust.edu.cn).
